# Prevalence of the cancer-associated germline variants in Russian adults and long-living individuals: using the ACMG recommendations and computational interpreters for pathogenicity assessment

**DOI:** 10.3389/fonc.2024.1420176

**Published:** 2024-09-05

**Authors:** Mariia Gusakova, Irina Dzhumaniiazova, Elena Zelenova, Daria Kashtanova, Mikhail Ivanov, Aleksandra Mamchur, Antonina Rumyantseva, Mikhail Terekhov, Sergey Mitrofanov, Liliya Golubnikova, Aleksandra Akinshina, Konstantin Grammatikati, Irina Kalashnikova, Vladimir Yudin, Valentin Makarov, Anton Keskinov, Sergey Yudin

**Affiliations:** The Federal State Budgetary Institution “Centre for Strategic Planning and Management of Biomedical Health Risks” of the Federal Medical Biological Agency, Moscow, Russia

**Keywords:** cancer, automated variant annotation, ACMG, germline variants, cancer genetic

## Abstract

**Background:**

Population studies are essential for gathering critical disease prevalence data. Automated pathogenicity assessment tools enhance the capacity to interpret and annotate large amounts of genetic data. In this study, we assessed the prevalence of cancer-associated germline variants in Russia using a semiautomated variant interpretation algorithm.

**Methods:**

We examined 74,996 Russian adults (Group 1) and 2,872 long-living individuals aged ≥ 90 years (Group 2) for variants in 28 ACMG-recommended cancer-associated genes in three steps: InterVar annotation; ClinVar interpretation; and a manual review of the prioritized variants based on the available data. Using the data on the place of birth and the region of residence, we determined the geographical distribution of the detected variants and tracked the migration dynamics of their carriers.

**Results:**

We report 175 novel del-VUSs. We detected 232 pathogenic variants, 46 likely pathogenic variants, and 216 del-VUSs in Group 1 and 19 pathogenic variants, 2 likely pathogenic variants, and 16 del-VUSs in Group 2. For each detected variant, we provide a description of its functional significance and geographical distribution.

**Conclusion:**

The present study offers extensive genetic data on the Russian population, critical for future genetic research and improved primary cancer prevention and genetic screening strategies. The proposed hybrid assessment algorithm streamlines variant prioritization and pathogenicity assessment and offers a reliable and verifiable way of identifying variants of uncertain significance that need to be manually reviewed.

## Introduction

Population studies are the foundation of epidemiology. Their outcomes guide public health decision-making. Cancer, as a leading cause of death worldwide, prompted multiple epidemiological studies of cancer prevalence and its risk factors.

Next-generation sequencing (NGS), such as whole genome sequencing (WGS) and whole exome sequencing (WES), has bolstered epidemiological studies with genetic data that has helped assess the prevalence of pathogenic variants, including those associated with cancer (1). However, there is no single approach that offers both accurate interpretation of WES and WGS data from large populations and optimizes the application of the criteria developed by the American College of Medical Genetics and Genomics (ACMG) (2). WES and WGS data often lead to the discovery of novel variants that may be widespread but have yet to be linked to cancer. These variants should be reported; however, in the absence of sufficient evidence and experimental confirmation of their nature, their accurate interpretation poses a challenge. Moreover, genetic data may show secondary, or incidental, findings that are not related to the primary purpose of testing but have clinical utility, making it even harder to interpret complex epidemiological data. It is crucial to provide an unbiased and reliable assessment of incidental findings and avoid their misclassification (3).

The ACMG recommendations for reporting incidental findings in clinical exome and genome sequencing seek to prevent overdiagnosis, avoid overloading clinical testing facilities, and provide an effective way to decrease cancer incidence and death rates. In 2015, ACMG and the Association for Molecular Pathology (AMP) jointly developed pathogenicity criteria that form accessible and clinically applicable guidelines and facilitate the clinical interpretation of complex genetic data (4). However, manually applying these criteria to large population datasets may be extremely complicated and prone to error. For instance, Amendola et al. assessed the performance of the ACMG-AMP Guidelines in nine laboratories. The guidelines were used in conjunction with the laboratories’ internal criteria. The interlaboratory concordance for both sets of criteria was 79% (K-alpha = 0.91), while the intralaboratory concordance for each individual set was only 34%. A total of 43 out of 194 (22%) patients exhibited differences in the categorical pathogenicity assessment, which may affect clinical decision-making. The application of the ACMG-AMP criteria resulted in a high frequency of tabulation errors. The authors concluded that using computational tools for variant classification offers a modest increase in application accuracy (5). With this in view, experts from the Clinical Genome (ClinGen), a resource funded by the National Institutes of Health (NIH), developed the ClinGen Pathogenicity Calculator (6).

The above criteria and variant interpretation tools are used worldwide for cancer studies (**Supplementary Table S1**). Huang et al. conducted the largest study of cancer predisposition variants (7). The authors found variants with single or cross-cancer associations and annotated variants of uncertain significance (VUSs). They also expanded the ACMG-AMP guidelines specific to rare cancer-associated variants and developed an automated variant classification pipeline called CharGer (Characterization of Germline Variants, https://github.com/ding-lab/CharGer). Jung Kim et al. used a semiautomated classifier, InterVar, that incorporates 10 of the 28 ACMG/AMP criteria (8). The authors classified SNVs in 24 cancer-associated genes on ACMG SF v2.0 and carried out a manual review of VUSs. Several studies have combined manual review and automated variant interpretation. However, very few of them offer a step-by-step description of the variant interpretation process. Most of them simply provide a list of detected variants without extensive supplementary materials.

Despite multiple cancer studies in different cohorts (**Supplementary Table S2**), the burden of hereditary cancer-associated variants in Russia has not been sufficiently assessed at either the population or individual level. This limitation applies to even cancer patients undergoing essential screening for a number of cancer syndromes. With this in mind, we sought to examine variants in the cancer-associated genes on the ACMG SF v3.0 list (for reporting of secondary findings in clinical exome and genome sequencing) (10) in a large representative sample of the Russian population. We chose a hybrid approach to variant interpretation, combining an ACMG criteria-based annotator, InterVar, an automated interpreter, ClinVar, and manual review. In this study, we refer to some of the VUSs as suspected to be pathogenic to stress that, despite their uncertain significance, they are highly concerning because they could be associated with cancer and should be given closer attention. One of the major outcomes of this study is the list of cancer-associated pathogenic and likely pathogenic variants (PVs and LPVs) and VUSs suspected to be pathogenic (del-VUSs). The list is supplemented with indications of the functional significance of the variants for protein biosynthesis. Moreover, we assessed the burden of cancer-associated germline variants in both adults and long-living individuals from all over Russia.

## Methods

### Recruitment of participants

Group 1 participants (n = 74,996) were randomly selected from 52 regions of Russia during the 2019–2022 epidemiological study on the prevalence of hereditary genetic variants associated with the risk of chronic diseases, including cancer. Group 2 participants (n = 2,872) were Moscow-based long-living adults aged 90 years and older (9).

General practitioner records (GP records) were available for all patients The GP records only provided information pertaining to the general health conditions of the participants, without specific indications on the course of the disease, if any. Cancer diagnoses with the ICD-10-CM codes (the International Classification of Diseases, 10th Revision, Clinical Modification) were known for 131 Group 1 participants, who had malignant neoplasms (ICD-10, С00–97). For Group 2, 174 participants had neoplasm/no neoplasm diagnoses with no corresponding ICD-10-CMs or tumor classifications (benign or malignant).

The study was approved by the ethics committee of the Centre for Strategic Planning of the Federal Medical and Biological Agency (protocol no. 5 from December 28, 2020, and protocol no. 2 from June 1, 2021). The participation of long-living adults and examination of their genetic predisposition were approved by the ethics committee of the Russian Clinical Research Center for Gerontology (protocol no. 30 from December 24, 2019). All participants provided informed consent to participate in the study. As part of the consent process, participants agreed to make their GP records available for review.

### Selection of genes and hereditary variants

The following 28 genes were analyzed: *APC*, *BMPR1A*, *BRCA1*, *BRCA2*, *MAX*, *MEN1*, *MLH1*, *MSH2*, *MSH6*, *MUTYH*, *NF2*, *PALB2*, *PMS2*, *PTEN*, *RB1*, *RET*, *SDHAF2*, *SDHB*, *SDHC*, *SDHD*, *SMAD4*, *STK11*, *TMEM127*, *TP53*, *TSC1*, *TSC2*, *VHL*, and *WT1* (10).

### Whole-genome sequencing and data processing

The QIAamp DNA Mini Kit (Qiagen, Germany) was used to isolate DNA from whole blood samples. The Nextera DNA Flex Kit (Illumina, USA) was used to prepare whole-genome sequence libraries, according to the manufacturer’s instructions. The samples were sequenced to 150 bp reads using the Illumina NovaSeq 6000 Sequencing System and S4 Reagent Kit (300 cycles) (Illumina, USA).

FASTQ files were obtained by demultiplexing the sequencing data in BCL format using Illumina bcl2fastq2 v2.20.0.422 Conversion Software. Illumina Sequencing Analysis Viewer v2.4.7 was used for sequencing quality control, and FastQC v0.11.9 was used for read quality control in FASTQ.GZ format. The reads were aligned to the reference genome, GRCh38.d1.vd1, using the Illumina DRAGEN Bio-IT Platform v07.021.510.3.5.7. The alignment quality of the BAM files was checked using DRAGEN FastQC v0.11.9, SAMtools v1.13, and mosdepth v0.3.1. All samples were checked for duplicates, unmapped reads, and other quality metrics. The mean sequencing coverage was 30x for all samples. Small variant calling of up to 50 bp was performed using Illumina Strelka2 v2.9.10 (11). Picard CrosscheckFingerprints with a pre-compiled haplotype map was used to check for duplicates.

### Analysis of the sequencing results in group 1

**Figure 1:**
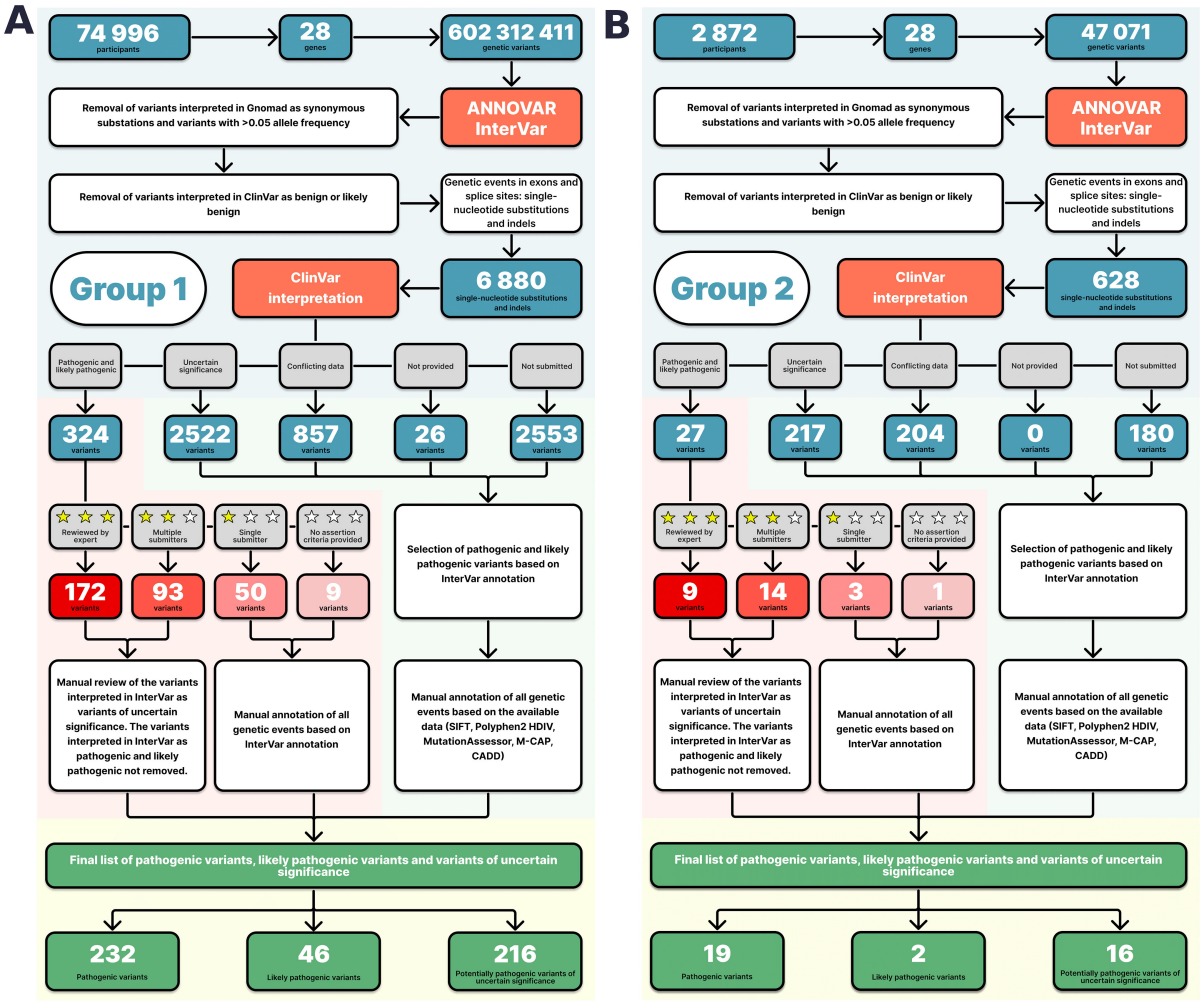
Pathogenicity interpretation algorithm for the Group 1 **(A)** and Group 2 **(B)** variants.

The sequenced data were analyzed for single-nucleotide variants, small insertions, and deletions (**Figure 1**). All the variants were annotated in InterVar (version 2021.07.27) (https://github.com/WGLab/InterVar) (12) that merges information from ClinVar, gnomAD, and *in silico* predictors (SIFT, Polyphen2 HDIV, MutationAssessor, REVEL, MetaLR, M-CAP, CADD, etc.) and applies 18 ACMG-recommended pathogenicity criteria. After filtration, synonymous substitutions and variants with an allele frequency of less than 5% (according to gnomAD) were removed. Only single-nucleotide substitutions, insertions, and deletions in exons and splice sites were further analyzed.

Variants annotated in ClinVar as benign or likely benign were removed. The resulting list contained InterVar-annotated variants, which were interpreted in ClinVar as PVs, LPVs, VUSs with conflicting data; and variants not previously submitted to ClinVar. The list included 6,880 SNVs, short indels, and substations at splice sites (**Supplementary Table S3**).

Further, the following ClinVar classifications were used:

I. Pathogenic or likely pathogenic (PV/LPV);

II. Variant of uncertain significance (VUS);

III. Conflicting data;

IV. Interpretation not provided;

V. Variant not submitted.

Variants annotated as PVs/LPVs in both ClinVar and InterVar with “expert panel” or “multiple submitters” review statuses were not further analyzed and were reported as PVs/LPVs. Variants interpreted in InterVar as variants of uncertain significance were further manually reviewed.

Variants interpreted as PVs/LPVs in ClinVar with a “single submitter” status or no review status were reviewed by experts based on their InterVar interpretations, available literature on clinical cases, results of *in silico* modeling, and *in silico* interpreter scores, such as SIFT <0.05, Polyphen2 HDIV ≥0,95, MutationAssessor ≥ 2, M-CAP >0,025, and CADD ≥15 (a consensus between three or more annotators was considered *in silico* evidence of likely pathogenicity; for variants with conflicting interpretations, REVEL >0.75 and MetaLR > 0.5 were used). The results of the analysis are presented in **Supplementary Table S4** in the Supplement. Variants interpreted as VUSs, variants with “conflicting data from submitters”, and variants “not provided” in ClinVar were further manually reviewed only if interpreted as PVs/LPVs in InterVar, following the above approach (**Supplementary Table S4**). **Figure 1A** shows the annotation diagram for Group 1.

### Analysis of the sequencing results in group 2

The data were analyzed using the above algorithm (**Figure 1B**; **Supplementary Tables S5, S6**).

### Comparative analysis of variants in groups 1 and 2

The prevalence analysis in both groups was based on the quantification of the allele frequency, the number of carriers, and the number of homozygotes and heterozygotes of the pathogenic variants and del-VUSs (**Supplementary Tables S7, S8**).

The comparative analysis was based on the assessment of the minor allele frequency (MAF) of PVs, LPVs, and del-VUSs in both groups. The MAF analysis generated a list of genetic variants common to Groups 1 and 2 (**Supplementary Table S9**).

### Analysis of the *RET* and *MAX* genes

Notably, *RET* (rearranged during transfection) and *MAX* (MYC Associated Factor X) are the only proto-oncogenes among tumor suppressor genes on the ACMG SF v3.0 list (for reporting of secondary findings in clinical exome and genome sequencing). In proto-oncogenes, the main cancer driving variants is gain-of-function (GoF), rather than loss-of-function (LoF). Therefore, variants with a proven or potential loss of function of the *RET* and *MAX*-encoded protein were not included in the general prevalence analysis (**Supplementary Table S7**).

The localization of LoF and GoF variants was analyzed both in within the gene and 3D protein structures. The structure of *RET* (ENST00000355710) was obtained from the Ensembl database. The model did not include intron sequences. The exons were equally spaced, and the introns were represented by symbols. The intron length distribution remained unchanged. The variant positions and the primary structure of the protein were obtained from the Single Nucleotide Polymorphism Database (dbSNP) and the UniProt database (2022/04 release), respectively. AlphaFold2 was used to predict the RET protein structure and to map the LoF and GoF variants. PyMOL was used for protein visualization.

### Analysis of the variant geographical distribution

The information on the place of birth and region of residence were obtained from the completed questionnaires (**Supplementary Data Sheet 1**). Assuming an uneven geographical distribution of the detected events, we mapped the nationwide migratory dynamics.

## Results

### The cohort

**Figure 2:**
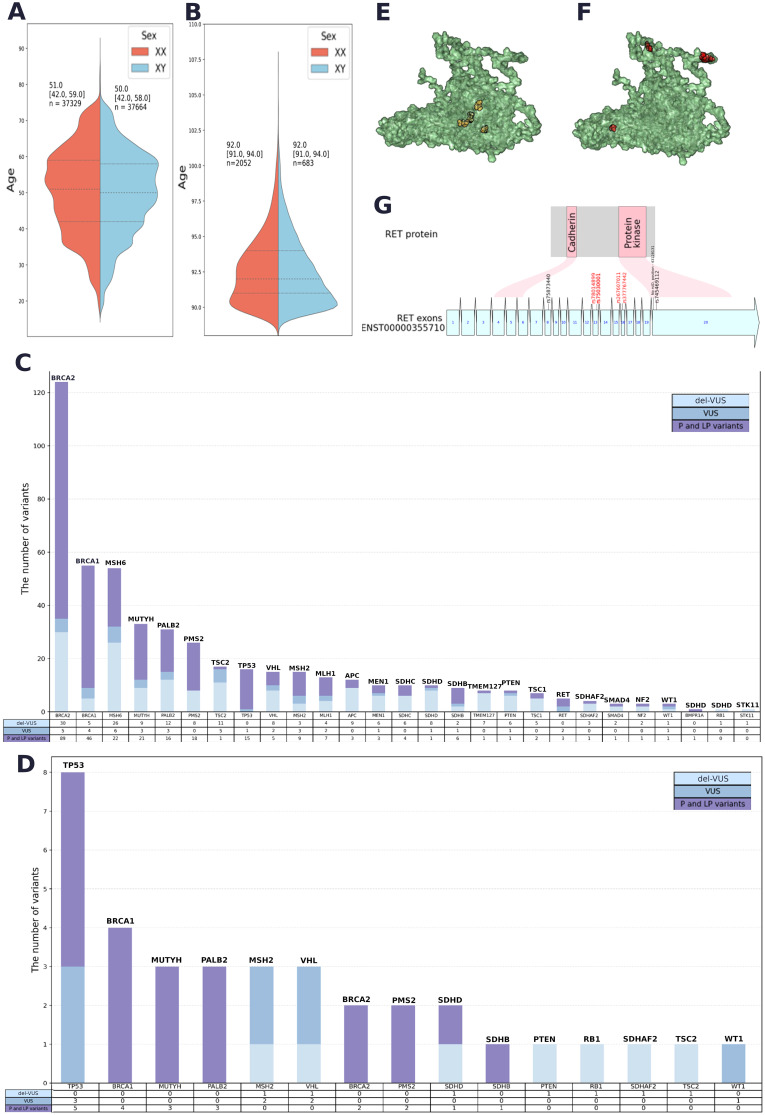
**(A)** Sex and age data for Group 1. **(B)** Sex and age data for Group 2. **(C)** Quantitative analysis of PVs, LPVs and del-VUSs within each gene in Group 1. **(D)** Quantitative analysis of PVs, LPVs and del-VUSs within each gene in Group 2. **(E)** Positions of GoF variants in a 3D model of the RET protein (AlphaFold 2). **(F)** Positions of LoF variants in a 3D model of the RET protein (AlphaFold 2). **(G)** Variant positions in *RET* and the corresponding protein domain. Red: GoF variants. Black: LoF variants associated with Hirschsprung’s disease.

Duplicates and potentially contaminated samples were removed. After the quality filtration, Group 1 included 74,996 participants aged 17–90 years (median age = 51 years for men and median age = 50 years for women) (**Figure 2А**). Group 2 included 2,872 long-living individuals aged ≥ 90 years who were not first-degree relatives (median age = 92 years) (**Figure 2B**); 71.4% of them were women.

### Analysis of variants in group 1

Whole-genome sequencing yielded 602,312,411 variants, with 1 411 232 variants shared between the 28 genes on the ACMG SF v3.0 list (for reporting of secondary findings in clinical exome and genome sequencing). The focus of the study was to examine prevalent pathogenic variants; therefore, synonymous substitutions, variants with an allele frequency of more than 5% (in gnomAD), and variants classified as benign or likely benign in ClinVar were not further analyzed. Only nonsynonymous substitutions and indels in exons and splice sites were further analyzed.

The resulting list contained 6,880 variants with ClinVar annotations and review statuses, InterVar pathogenicity interpretations, and *in silico* interpretations (see Methods) (**Supplementary Table S3**). A total of 232 PVs, 46 LPVs, and 216 del-VUSs (**Supplementary Table S4**). All PVs and LPVs were detected within 1,743 genomes. The largest number of both types of variants were found in *BRCA2, BRCA1, MSH6, MUTYH, PMS2, PALB2, TP53*, and *MSH2* (**Figure 2C**).

Among prioritized VUSs interpreted as LPVs in both automated and manual reviews, we detected 175 novel del-VUSs that had not been previously reported or submitted to ClinVar. Only four of them had been previously submitted to gnomAD. Del-VUSs were found in the following genes: *STK11, TSC2, PMS2, VHL, SDHB, PALB2, NF2, BRCA2, WT1, MLH1, BRCA1, MUTYH, MSH2, MSH6, RB1, SMAD4, SDHAF2, MEN1, PTEN, TMEM127, SDHD, TSC1*, and *SDHC* (**Figure 2C**).

### Analysis of variants in group 2

Group 2 analysis yielded 1,148,036 unique variants. A total of 49,071 variants were shared between the 28 genes on the ACMG SF v3.0 list (for reporting of secondary findings in clinical exome and genome sequencing). The detected variants were analyzed following the established algorithm (**Figure 1B**). A total of 21 pathogenic and likely pathogenic SNVs and indels and 16 del-VUSs (**Supplementary Table S8**) were selected from 628 variants with ClinVar annotations and review statuses, InterVar interpretations, and *in silico* interpretations (**Supplementary Table S7**).

The qualitative analysis (**Figure 2D**) showed that *TP53, BRCA1, MUTYH, PALB2, BRCA2*, and *PMS2* had the largest number of PVs and LPVs, while *TP53, MCH2*, and *VHL* had the largest number of del-VUSs. The detected trends in Groups 1 and 2 were similar but not identical: certain genes that were widely prevalent in Group 1, such as *MSH6*, were not detected in Group 2.

### Comparative analysis of the lists of variants in groups 1 and 2

Twenty-two PVs and LPVs, and del-VUSs were detected in 860 Group 1 participants and 38 Group 2 participants (**Supplementary Table S9**). Most of these variants were found in *MUTYH*. The variants in *BRCA1* and *PMS2* have “reviewed by expert panel” status, and variants in *MUTYH, PMS2, VHL, PALB2*, and *SDHB* have “multiple submitters” status.

The WGS showed that 1.82% of participants in Group 1 carried PVs/LPVs in 28 ACMG genes. Surprisingly, 1,98% of participants in Group 2—long-living individuals expected to carry very few cancer-associated genetic variants—carried PVs/LPVs in 8 ACMG genes; 15 out of the 37 detected variants had “reviewed by expert panel” and “multiple submitters” statuses in ClinVar.

### Quantitative and qualitative analyses of variants in groups 1 and 2

We also analyzed the prevalence of functional types. First, the detected variants were classified based on their impact on the protein sequence (**Table 1**). Group 1 demonstrated an absolute prevalence of stop-gain variants, while Group 2 had mostly nonsynonymous substitutions.

**Table 1:** Classification of PVs, LPVs and del-VUSs in Groups 1 and 2, according to their impact on protein sequence.

Variant annotation in RefGene	PVs and LPVs registered in ClinVar	Del-VUSs registered in ClinVar	Novel del-VUSs
Group 1 (N=74 996)			
**Nonsynonymous SNV**	63	17	6
**Stop-gain**	94	0	45
**Frameshift deletion**	75	13	62
**Frameshift insertion**	22	3	40
**Splice sites**	24	8	27
Group 2 (N=2 872)			
**Nonsynonymous SNV**	9	5	0
**Stop-gain**	5	1	3
**Frameshift deletion**	5	2	2
**Frameshift insertion**	1	0	2
**Splice sites**	1	0	1

The number of functional variants in each gene was calculated based on the RefGene annotations (**Table 2**). The probability of being loss-of-function-intolerant (pLI) and a continuous measure of loss-of-function observed/expected upper bound fraction (LOEUF) were obtained from gnomAD v2.1.1 (pLI > 0.9, indicating intolerance; LOEUF <0.35). These scores reflect a gene’s tolerance to loss of function. Protein-truncating variants (PTVs) are one of several mechanisms (along with mRNA isoforms and alternative translation) by which premature termination codons (PTCs) are introduced into transcripts. It is widely known that PTCs are likely to be targeted by processes aimed at reducing errors in gene expression, which is believed to lead to a predicted loss-of-function (LoF) variant or null allele. A high LOEUF indicates a relatively high tolerance to inactivating variants, while a low LOEUF indicates rigorous selection against loss-of-function variation.

**Table 2:** RefGene-based functional annotations of pPVs and LPVs in Group 1.

Gene	Nonsynonymous SNV	Stop-gain	Frameshift deletion	Frameshift insertion	Splice sites	pLI, gnomAD v2.1.1	LOEUF, gnomAD v2.1.1*	Total per-gene number of alleles in PVs, LPVs, and del-VUSs		Total Number of alleles in conserved and nonconserved genes	
								Group 1	Group 2	Group 1	Group 2
TSC2	0	0	1	0	0	1	0.074	17	1	130	6
NF2	0	0	0	0	1	1	0.086	3			
TSC1	1	1	0	0	0	1	0.118	7			
RET	3	0	0	0	0	1	0.123	6			
RB1	0	0	0	0	0	1	0.126	0	1		
APC	0	3	0	0	0	1	0.161	14			
MEN1	3	0	0	0	0	1	0.171	18			
SMAD4	1	0	0	0	0	1	0.222	5			
STK11	0	0	0	0	0	0.99	0.245	1			
WT1	1	0	0	0	0	1	0.247	10	1		
MSH2	1	4	3	0	1	0.9	0.334	48	3		
BMPR1A	0	1	0	0	0	0.9	0.335	1			
MAX	0	0	0	0	0	0.83	0.462	0		1534	48
TP53	15	0	0	0	0	0.53	0.469	20	8		
MSH6	3	10	5	3	1	0	0.498	77			
PTEN	0	0	0	0	1	0.26	0.507	10	1		
MLH1	3	3	0	0	1	0	0.575	20			
BRCA2	8	31	33	12	5	0	0.635	250	3		
SDHD	1	0	0	0	0	0.34	0.731	10	3		
SDHB	1	2	0	0	3	0	0.825	21	1		
BRCA1	4	15	19	4	4	0	0.915	140	6		
VHL	3	2	0	0	0	0.08	0.927	70	3		
PALB2	0	6	8	0	2	0	1.006	127	3		
TMEM127	0	0	1	0	0	0,01	1.154	12			
MUTYH	11	7	1	1	1	0	1.191	738	17		
SDHC	2	1	0	0	0	0	1.256	13			
PMS2	2	7	4	2	3	0	1.266	26	3		

*Table presents ranked lists of genes based on their LOEUFs.

In Group 1, *TSC2, NF2, TSC1, RET, RB1, APC, MEN1, SMAD4, STK11*, and *WT1* had the highest pLI (**Table 2**). As expected, genes with the largest number of pathogenic variants, such as *PMS2* and *MUTYH*, had the lowest pLI (**Table 2**) and the highest number of stop-gains and nonsynonymous substitutions. We could not detect a clear gene-specific tendency in Group 2. According to our analysis of their pLIs and LOEUFs, genes with PVs, LPVs and del-VUSs were not conserved, except for *MSH2*, which had borderline values. **Table 2** shows the comparison of the overall number of substitutions in conserved (high pLI) and nonconserved (low pLI) genes. In both groups, most of the alleles occurred in less conserved LoF-tolerant genes with a LOEUF index of greater than 0.35 (gnomAD) (**Table 2**). **Supplementary Table S4** (Supplements) presents the complete list of the RefGene functional annotations of del-VUSs in Group 1.

The frequency analysis showed that sixteen variants had an MAF ≥ 0.0001 (**Table 3**). As expected, *MUTYH* associated with recessive familial adenomatous polyposis, the only two homozygotes of which also carry biallelic *MUTYH* variants, had the highest allele frequency, followed by *VHL*, *BRCA1*, *BRCA2*, and *PALB2* (**Supplementary Tables S7, S8**).

**Table 3:** Variants with an MAF of ≥ 0,0001 in 74,996 Group 1 participants.

Gene	Interpretation	Variant ID	HGVS	Number of alleles in Group 1	Number of heterozygotes in Group 1	Number of homozygotes in Group 1	MAF in Group 1	gnomAD
MUTYH	Likely Pathogenic	rs36053993	NC_000001.11:g.45331556C>T	441	439	2	0.0052	0.0032
MUTYH	Likely Pathogenic	rs34612342	NC_000001.11:g.45332803T>C	113	113	0	0.00075	0.0015
MUTYH	Likely Pathogenic	rs140342925	NC_000001.11:g.45332445C>T	92	92	0	0.00061	9.701e-05
VHL	Likely Pathogenic	rs1346312258	NC_000003.12:g.10142957T>C	49	49	0	0.00033	.
PALB2	Pathogenic	rs180177102	NC_000016.10:g.23634957del	27	27	0	0.00018	9.684e-05
SDHD	VUS	rs104894302	NC_000011.10:g.112089002A>G	26	26	0	0.00017	.
PALB2	Pathogenic	rs515726123	NC_000016.10:g.23636037_23636038del	25	25	0	0.00017	.
BRCA1	Pathogenic	rs28897672	NC_000017.11:g.43106487A>C	23	23	0	0.00015	.
MSH2	VUS	rs1194793421	NC_000002.12:g.47414419del	22	22	0	0.00015	.
PALB2	Pathogenic	rs180177143	NC_000016.10:g.23637886_23637887del	20	20	0	0.00013	6.457e-05
PMS2	VUS	rs200029834	NC_000007.14:g.6002670G>C	18	18	0	0.00012	0.0002
BRCA2	Pathogenic	rs746229647	NC_000013.11:g.32338202_32338203del	17	17	0	0.00011	3.237e-05
BRCA2	Pathogenic	rs80358754	NC_000013.11:g.32339641T>G	17	17	0	0.00011	.
BRCA1	Pathogenic	rs80357711	NC_000017.11:g.43091497del	16	16	0	0.00011	0.0002
MUTYH	VUS		NC_000001.11:g.45331234C>T	15	15	0	0.0001	.
MUTYH	Pathogenic	rs587780088	NC_000001.11:g.45334493G>A	15	15	0	0.0001	.

We conducted a separate analysis for the cancer-associated genes *RET* and *MAX* (**Supplementary Table S5**). We compared the locations of these substitution sites within the protein. We detected the most significant difference in the *RET* gene product (**Figures 2E, F**, and G): gain-of-function (GoF) variants and loss-of-function (LoF) variants tended to occupy different positions in *RET*—GoF substitutions were localized in the center of the protein (**Figure 2E**), while LoF substitutions were “scattered” on the periphery (**Figure 2F**). Moreover, GoF substitutions were located outside of the functional regions of the protein, while two LoF substitutions were located in the domain that has protein kinase activity (**Figure 2G**).

### Diseases associated with the detected genetic variants

The most prevalent variants in Group 1 were associated with hereditary breast and ovarian cancer, Lynch syndrome, *MUTYH*-associated polyposis, Li-Fraumeni syndrome, and Tuberous sclerosis complex. A similar trend was observed in Group 2 (**Supplementary Table S11**).

In Group 1, none of the 31 participants with malignant neoplasms carried hereditary variants. In Group 2, four out of 174 participants with neoplasms carried variants in *BRCA2* (rs768580992, review status “reviewed by expert panel”) and MUTYH (rs36053993, rs36053993, and rs34612342; review status “multiple submitters”).

The GP records of the cancer patients did not specify the cancer type or tumor classification (benign or malignant). Notably, none of the 31 participants in Group 1 with cancer carried cancer-predisposing genes. Pathogenic variants in *BRCA2* and *MUTYH* with “reviewed by expert panel” and “multiple submitters” statuses were detected in four out of 174 participants.

### Analysis of the geographical distribution

**Figure 3:**
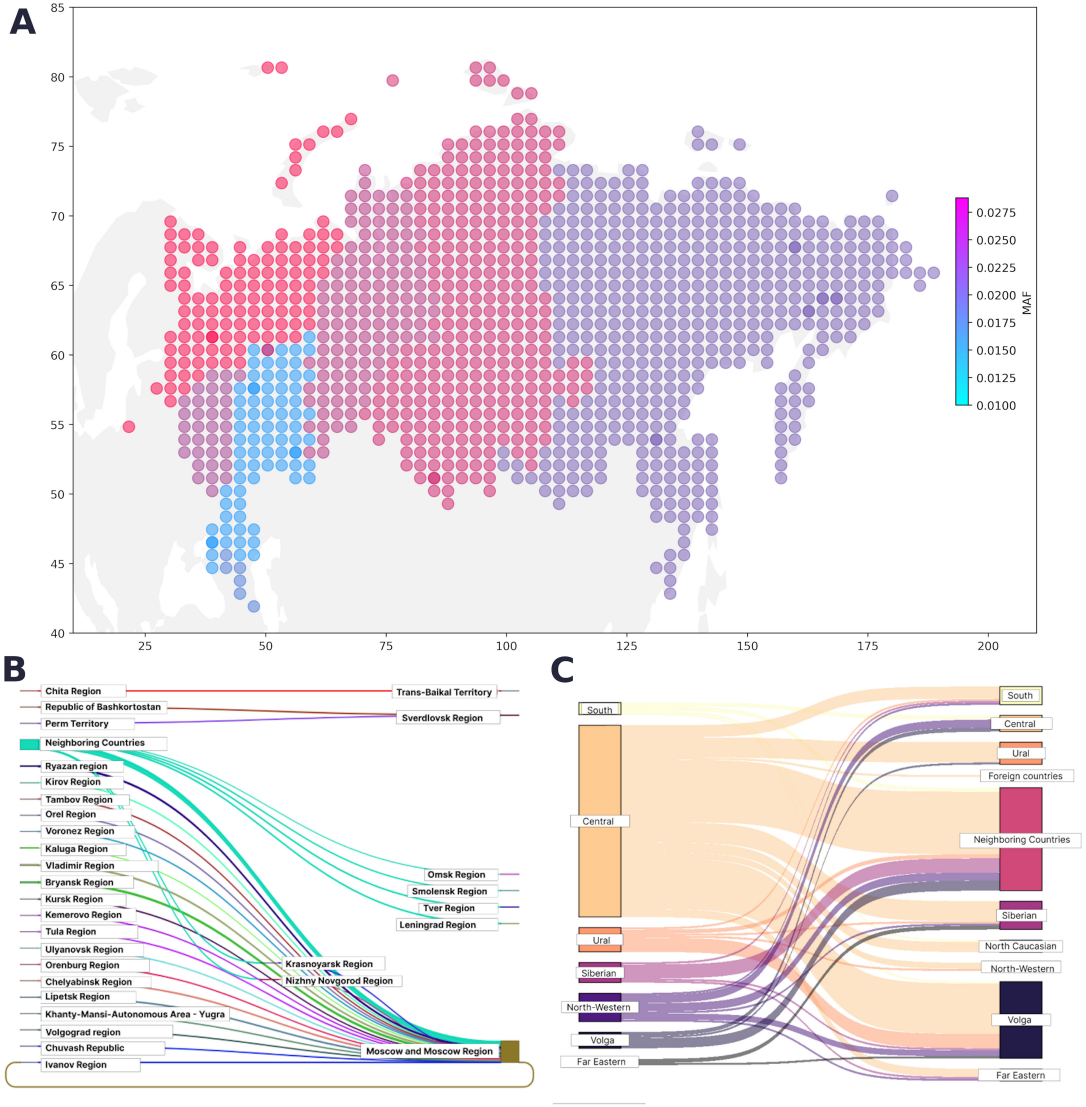
**(A)** Geographical distribution of PVs, LPVs, and del-VUSs. The color represents the minor allele frequency in the region; the color intensity corresponds to an increase in the proportion of variant carriers. **(B)** Map of migration dynamics in variant carriers (federal districts). **(C)** Map of migration dynamics in variant carriers (region).

We assessed the geographical distribution of the pathogenic cancer-associated variants as the ratio of the overall population in the region to the number of variant carriers in that region (**Figure 3A**). The largest number of pathogenic variants was found in Moscow and the Central Federal District, followed by the Urals and Siberia. The smallest number of cancer-associated pathogenic variants was found in the Caucasus and nearby regions.

The region of residence and the place of birth was known for 31,842 participants from the entire cohort. The regions in our study were represented unevenly, making it challenging to assess the true contribution of geographic location. However, we determined the proportion of variant carriers in each region. **Figures 3B** and 3C show the maps of the migration dynamics. Moscow and the Moscow oblast had the largest number of carriers, which may be attributed to the participant recruitment strategy.

## Discussion

In this study, we report the results of the largest assessment of the prevalence of germline cancer syndrome-associated variants in Russian adults and long-living individuals. This is also the first Russian study based on the ACMG recommendations for reporting incidental, or secondary, findings in clinical exome and genome sequencing (10).

We observed a consistent pattern of highly LoF-tolerant genes containing a greater variety of variants. Expectedly, we did not detect any PVs/LPVs in the nontolerant genes in the long-living individuals. In Group 1, an absolute allele-per-gene leader was the single recessive gene *MUTYH* with a 2-homozygous rs36053993 variant, followed by *VHL*, *PALB2*, *SDHD*, *PALB2*, *BRCA1*, *MSH2*, *PALB2*, *PMS2*, *BRCA2*, *BRCA1*, and *MUTYH* (carriers > 10; MAF > 0.0001) in both Group 1 and Group 2. In the study by Huang et al., *BRCA1*, *BRCA2*, *ATM*, *PALB2*, *RET*, *NF1*, *MSH6*, *TP53*, and *VHL* were the most common genes containing germline PVs/LPVs in 10,389 adult cancer patients (7). In children, more variants were found in LoF-intolerant genes. In the study by Zhang et al., the most prevalent mutant genes in children with cancer were *TP53*, *APC*, *BRCA2*, *NF1*, *PMS2*, *RB1*, and *RUNX1* (13). *BRCA1*, *BRCA2*, *PALB2*, *TP53*, *MSH6*, and *PMS2* are frequently reported as mutated variants across all groups (healthy adults/children, adults/children with cancer, and long-living individuals). Therefore, it is crucial to further investigate the effect of each individual genetic event to ensure appropriate prevention and/or therapeutic strategies.

Many authors have reported that *in silico* predictors tend to recognize LoF variants better than deleterious GoF variants and often assign extreme values to the former and average values to the latter (14, 15). However, the manual review showed that this fact does not compromise the reliability of the decision-making process. Moreover, in Group 1, we detected and analyzed both types of variants in the *RET* gene, which is widely known to be associated with cancer. There is evidence to suggest that LoF and GoF variants in structural variants do not reside in the same locations within a gene or its protein domains (26). We observed this tendency in the *RET* gene and its corresponding protein: the GoF substitutions were localized in the “core’ of the protein, while the LoF substitutions were located on its “outskirts”.

People aged 90 years and older are unlikely to carry highly penetrant pathogenic variants in cancer-associated genes with autosomal dominant inheritance. The variants detected in this group of participants are likely to have lower penetrance, even the highly pathogenic ones. The factors that contribute to their expression as a disease phenotype require further research. The PVs, LPVs and del-VUSs found in these participants are not supported by substantial clinical evidence and should be reviewed by geneticists. The available publications report similar results and conclusions. Pinese et al. conducted a study in 2,570 Australian older adults and found that the cohort had fewer pathogenic genetic variants than the UK Biobank and gnomAD cohorts. However, 28 participants carried LPVs in genes recommended for reporting by the ACMG, including the cancer-associated genes *BRCA2*, *MSH2*, *MSH6*, and *PMS2* (16). Zheng et al. studied 51 older adults from families whose “cancer-free” status had been confirmed in approximately 1000 blood relatives. The authors did not detect any PVs or LPVs (17).

Here, we report novel VUSs that have not been previously submitted to ClinVar, which we suspect to be pathogenic based on *in silico* interpretations (**Supplementary Table S4**) and provide an assessment of the prevalence of genes with this type of substitution in the Russian population. The following genes had the largest number of del-VUSs: *BRCA2* (30 variants), *MSH6* (26 variants), *PALB2* (12 variants), and *TSC2* (11 variants). Notably, these novel VUSs are mainly “stop-gain”, “frameshift deletion”, “frameshift insertion”, or “splice sites” variants and are known as protein-truncating variants (PTVs). These variants are likely to have a serious negative impact on the function of the encoded protein. Therefore, they are critical for interpreting genomic data and building therapeutic hypotheses. InterVar automatically assigned “PVS1” to PTVs based on its own functional gene annotation criteria and database of loss-of-function intolerant genes (12). Only 46 novel SNVs suspected to be pathogenic were assigned “PVS1 = 0” (**Supplementary Table S4**). As explicitly stated by the ACMG/AMP (4), the detection of PTVs is insufficient for a conclusive interpretation of the pathogenicity of a variant, particularly novel del-VUSs found in population studies. Additional functional analysis of the mRNA and proteins is required to interpret “nonsense”, “frameshift”, “canonical +/−1 or 2 splice site”, and “exon-level deletion” variants as null variants. Karczewski et al. noted that variants annotated as “loss of function” tend to trigger false *in silico* interpreter responses more often than synonymous or other benign variants (18). In our manual review, we focused on the available clinical case records and family histories and/or modeling results of the pathogenic effects on the mRNA or protein. This could account for the interpretation of a number of variants as del-VUSs. For instance, 175 variants were submitted to ClinVar as PVs or LPVs with “single submitter” status and interpreted in InterVar as PVs or LPVs. However, after manual review, 50 of these VUSs were reinterpreted as del-VUSs. We reinterpreted all novel variants classified as PVs/LPVs in InterVar as del-VUSs, due to the lack of available data to support or refute our interpretation.

Hirschsprung’s disease caused by LoF variants in *RET* was not the focus of this study; however, we report three novel variants interpreted as del-VUSs: NC_000010.11:g.43128194A>T, NC_000010.11:g.43128118C>A, and NC_000010.11:g.43128131A>G.

Our findings did not indicate a definitive link between carrying PVs/LPVs and cancer, suggesting a lower penetrance and an extremely late clinical expression of these variants. They also serve as another confirmation of the sporadic nature of cancer, the development and manifestation of which are influenced by a whole spectrum of genetic and nongenetic factors. Additionally, the presence of heterozygous pathogenic variants alone may not be sufficient for cancer development, which could explain the lack of a definitive link in our study.

The geographical prevalence of variants in Group 1 was uneven; therefore, it was difficult to find statistically significant associations between geographic location and allele frequency. We observed more variant carriers in regions with a higher population density and large metropolitan areas, which could be attributed to migration. From a healthcare standpoint, knowing the geographic distribution of cancer-associated variants is critical, particularly in regions with a high rate of carriers, and is integral to providing proper care and preventing healthcare system overload.

In conclusion, it is important to highlight the benefits offered by automated variant annotation for genomic cancer studies, as demonstrated in this study. Population genomic studies provide large amounts of data, including incidental findings, and help discover novel clinically significant genetic variants. In this study, we report novel del-VUSs. These del-VUSs have not been previously submitted to ClinVar and were detected owing to the advantages offered by the automated annotator InterVar, which generates variant interpretations from *in silico* predictors based on the ACMG criteria and population databases, such as gnomAD and RefGene. This allowed us to select and prioritize genetic variants for manual annotation. As shown in **Figure 1**, InterVar was critical for the selection of variants that had been submitted to ClinVar as variants of unknown significance (4,421 SNVs and indels). We selected only variants classified by InterVar as P/LP, which reduced the number of variants for manual interpretation to 64. It took the expert approximately 30 to 90 minutes to interpret a single variant. This serves to demonstrate that automated tools are essential for the interpretation of incidental findings in large population cohorts, which differs from the interpretation of a single clinical case with a known disease phenotype, known medical history, and readily available genetic testing of the patient’s relatives.

## Conclusion

The present study used a systematic and rigorous approach consistent with the best clinical practices. This approach enabled us to assess the burden of cancer-associated hereditary variants in the Russian population, determine the geographical distribution of the carriers of the detected variants, and track their migration dynamics. The findings of this study could contribute to the development of new prevention and genetic screening strategies. The proposed variant assessment algorithm offers a time-efficient and easy method for variant prioritization and interpretation of large amounts of genetic data. It also streamlines the pathogenicity assessment of variants of uncertain significance that may contribute to a genetic predisposition to cancer.

## Limitations

More research is needed to collect enough RNA expression data and other experimental data, which could confirm or disprove the functional annotation of the variants as “gain-of-function” or “loss-of-function” variants, as well as VUSs status.

The variant interpretations presented in this study reflect the authors’ conclusions and do not constitute validated variant annotations for clinical reporting. Variant validation using Sanger sequencing is currently underway.

We found that the vast majority of the truncated variants trigger InterVar’s PVS1 rule (very strong criterion) and are interpreted as pathogenic. InterVar interpreted individual variants with “expert panel” status as VUSs. These variants should be investigated and, possibly, reannotated. InterVar performs best for variants with “single submitter” or “no submitter, criteria provided” status. It interpreted certain pathogenic variants as VUSs, which was instrumental in drawing experts’ attention to these variants for further manual review. However, InterVar generated a number of erroneous interpretations and cannot replace manual review.

## Data Availability

Variant analysis conducted during the current study is fully available within the paper and its **Supplementary Materials**. The data that support the findings of this study are not openly available due to reasons of sensitivity and are available from the corresponding author upon reasonable request. Data are located in controlled access data storage at The Federal State Budgetary Institution “Centre for Strategic Planning and Management of Biomedical Health Risks” of the Federal Medical Biological Agency.
