# Supplementary material for: Prevalence of the cancer-associated germline variants in Russian adults and long-living individuals: using the ACMG recommendations and computational interpreters for pathogenicity assessment
Source: Front Oncol. 2024 Sep 5;14:1420176. doi: 10.3389/fonc.2024.1420176 (PMC11410565; doi:10.3389/fonc.2024.1420176)
Supplement: Supplementary file 1 [file DataSheet1.zip › Supplementary Data Sheet 1.DOCX]

**Data S1. Participant questionnaire.**

1. Participant ID:

2. Sex: 1 – male, 2 – female.

3. Date of birth: ⌴⌴.⌴⌴. ⌴⌴⌴⌴ y.

4. Place of birth indicated in the national passport:

5. Region of residence:

6. History of illness (with ICD10 code):

7. Date of examination: ⌴⌴.⌴⌴ .20__г.

8. Date of blood collection ⌴⌴.⌴⌴. 20__г.

Name of the Doctor:_____________________________ Signature:__________________________
