# Supplementary material for: Prevalence of the cancer-associated germline variants in Russian adults and long-living individuals: using the ACMG recommendations and computational interpreters for pathogenicity assessment
Source: Front Oncol. 2024 Sep 5;14:1420176. doi: 10.3389/fonc.2024.1420176 (PMC11410565; doi:10.3389/fonc.2024.1420176)
Supplement: Supplementary file 1 [file DataSheet1.zip › Supplementary Table 1.DOCX]

**Table S1. Studies based on the ACMG criteria.**

| **Citation** | **Population** | **Sample size and participant profiles** | **Percentage of detected pathogenic or likely pathogenic variants based on the ACMG criteria** | **Number of pathogenic and likely pathogenic germline mutations** |
| --- | --- | --- | --- | --- |
| [9] | UK Biobank British Irish cohort, African, and South Asian cohorts | 149,960 individuals from | 4,1% (WGS, ACMG v3.0, 2021);  2,0% (based on WES, ACMG v2.0  2.5% (based on WGS, ACMG v2.0)  . | - |
| [10] | East Asians | 954 | 2,5% (WGS, ACMG v2.0) | 21 |
| [11] | Arab population from Qatar | 88 (WGS) +  917 (WES) | 0,6% (ACMG list v2.0) | 4 |
| [12] | Koreans | 100 healthy controls  96 patients with suspected genetic disorders | 6,63% (ACMG list, 2013)  7% (Control group)  6% (Hereditary diseases cohort) | 11 |
| [13] | Caucasian ethnicities (91%)  Non-Caucasian ethnicities (9%) | 836 patients with non-obstructive azoospermia | 3,3 % (ACMG list v2.0) | - |
| [14] | Australians | 1. 2570 elderly Australians depleted for cancer | 0,4% (ACMG list v2.0) | 10 |
