# Supplementary material for: Prevalence of the cancer-associated germline variants in Russian adults and long-living individuals: using the ACMG recommendations and computational interpreters for pathogenicity assessment
Source: Front Oncol. 2024 Sep 5;14:1420176. doi: 10.3389/fonc.2024.1420176 (PMC11410565; doi:10.3389/fonc.2024.1420176)
Supplement: Supplementary file 1 [file DataSheet1.zip › Supplementary Table 11.DOCX]

**Table S11.** Cancer-associated syndromes ranked by the number of pathogenic and likely pathogenic variants in Groups 1 and 2.

| **Cancer-associated syndromes** | **Number of pathogenic and likely pathogenic variants per syndrome in Group 1** | **Number of alleles per syndrome in Group 1** | **Number of pathogenic and likely pathogenic variants per syndrome in Group 2** | **Number of alleles per syndrome in Group 2** |
| --- | --- | --- | --- | --- |
| Hereditary breast and/or ovarian cancer | 151 | 432 | 9 | 12 |
| Lynch syndrome | 56 | 171 | 2 | 2 |
| MUTYH-associated polyposis | 21 | 705 | 3 | 17 |
| Li–Fraumeni syndrome | 15 | 18 | 5 | 5 |
| Hereditary paraganglioma–pheochromocytoma syndrome | 13 | 64 | 2 | 2 |
| von Hippel–Lindau syndrome | 5 | 56 | 0 | 0 |
| Multiple endocrine neoplasia type 1 | 4 | 18 | 0 | 0 |
| Tuberous sclerosis complex | 4 | 24 | 0 | 0 |
| Familial adenomatous polyposis | 3 | 3 | 0 | 0 |
| Familial medullary thyroid cancer | 3 | 3 | 0 | 0 |
| Juvenile polyposis syndrome | 2 | 2 | 0 | 0 |
| Neurofibromatosis type 2 | 1 | 1 | 0 | 0 |
| PTEN hamartoma tumor syndrome | 1 | 1 | 0 | 0 |
| WT1-related Wilms tumor | 1 | 1 | 0 | 0 |
| Peutz–Jeghers syndrome | 0 | 0 | 0 | 0 |
| Retinoblastoma | 0 | 0 | 0 | 0 |
