# Supplementary material for: Prevalence of the cancer-associated germline variants in Russian adults and long-living individuals: using the ACMG recommendations and computational interpreters for pathogenicity assessment
Source: Front Oncol. 2024 Sep 5;14:1420176. doi: 10.3389/fonc.2024.1420176 (PMC11410565; doi:10.3389/fonc.2024.1420176)
Supplement: Supplementary file 1 [file DataSheet1.zip › Supplementary Table 2.DOCX]

**Table S2. Studies on the frequency of hereditary cancer-associated variants in adults and children.**

| **Citation** | **Population** | **Sample size and participant profiles** | **Percentage of carriers of the cancer-associated variants** | **Number of pathogenic and likely pathogenic cancer-associated germline mutations** |
| --- | --- | --- | --- | --- |
| [7] | European cohort | 1. 10,389 adults with 33 different cancer types (TCGA) | 8% | 853 |
| [16] | East Asian cohort | 1. 2401 tumor-matched normal samples across 20 types of cancer and 2 precancerous lesions (COGVIC) | 9,7% | 233 |
| [15] | USA | 1. family-based cancer research cohort (n = 1173) | 1,2% | 11 |
|  |  | 1. Сancer-free ethnicity-matched controls (n = 982) | 0,8% | 8 |
| [17] |  | 1. 51 elderly cancer-free individuals, whose numerous (ca. 1000) family members were found to be cancer-free (‘cancer-free families’, CFFs) based on face-to-face interviews | 0% | 0 |
| [18] | USA | 1. 1120 patients younger than 20 years of age | 8,5% | 95 |
|  |  | 1. 966 persons who did not have known cancer in the 1000 Genomes Project | 1,1% | 10 |
|  |  | 1. data from an autism study (from 515 persons with autism and 208 persons without autism | 0,6% | 4 |
| [19] |  | 1. 1507 children, adolescents, and young adults with solid tumors | 12% | 180 |
